# Golgi-derived phosphatidylinositol 4-phosphate is crucial for organization of the preautophagosomal structure

**DOI:** 10.1016/j.jbc.2026.111445

**Published:** 2026-04-09

**Authors:** Huichao Lang, Kuninori Suzuki

**Affiliations:** 1 Department of Integrated Biosciences, Graduate School of Frontier Sciences, The University of Tokyo, Kashiwa, Chiba, Japan; 2 Life Science Data Research Center, Graduate School of Frontier Sciences, The University of Tokyo, Kashiwa, Chiba, Japan; 3 Collaborative Research Institute for Innovative Microbiology, The University of Tokyo, Bunkyo, Tokyo, Japan

**Keywords:** Atg9, autophagosome biogenesis, isolation membrane, lipid transfer, phosphatidylinositol 4-phosphate, Pik1, *Saccharomyces cerevisiae*

## Abstract

Macroautophagy (hereafter autophagy) is a conserved intracellular degradation pathway that is essential for maintaining cellular homeostasis. Autophagosome formation involves preautophagosomal structure (PAS) organization and expansion of the isolation membrane (IM). Although phosphatidylinositol 4-phosphate (PtdIns4P) localizes to the IM and is required for autophagy, the specific functional role it plays in autophagosome formation remains unclear. Pik1, a PtdIns 4-kinase localized to the Golgi, plays a critical role in this process. We used temperature-sensitive *pik1* mutant cells and found that PAS localization of autophagy-related Atg9, Atg17, Atg1, and Atg13 remained normal at the restrictive temperature, indicating that PAS scaffold formation was unaffected. In contrast, the recruitment of downstream Atg proteins, the PtdIns 3-kinase complex I, including Atg14, the Atg2–Atg18 complex, and Atg8, was impaired under the same condition. These findings demonstrate that Pik1-generated PtdIns4P is essential for PAS organization. Since Atg9 vesicles are derived from the Golgi, we hypothesized that PtdIns4P is transported to the PAS on Atg9 vesicles to mediate recruitment of downstream Atg proteins. To test this, we performed immunoprecipitation analysis using a PtdIns4P-binding protein and found that Atg9 was coimmunoprecipitated at the permissive temperature but not at the restrictive temperature. This result indicates that PtdIns4P is enriched on Atg9 vesicles through Pik1 activity. Moreover, our assessment in *pik1* mutant cells showed that IM expansion is impaired at the restrictive temperature. Collectively, these results identify PtdIns4P as a key component of PAS organization.

Macroautophagy (hereinafter abbreviated as "autophagy") is an important physiological process that requires the formation of a double-membrane structure enclosing sequestered cytoplasmic material, the autophagosome (AP). Autophagy was once described merely as an intracellular degradation system necessary for cellular homeostasis (1, 2); however, in recent years, its dysregulation has been linked to various human diseases, including cancer and neurodegenerative disorders, and its artificial modulation is now considered a promising therapeutic strategy (3, 4, 5, 6).

Autophagy proceeds through several sequential stages, beginning with induction, followed by preautophagosomal structure (PAS) organization, expansion of the isolation membrane (IM), maturation of the AP, and finally degradation of the enclosed cargo (7, 8). In the yeast *Saccharomyces cerevisiae*, autophagy induction triggers the accumulation of autophagy-related (Atg) proteins on the vacuolar membrane to form the PAS in close proximity to the endoplasmic reticulum (7, 8). The IM then develops and sequesters cytoplasmic materials, and its ends close to generate the double membrane–bound AP. After APs fuse with the vacuole, their contents are degraded within the vacuolar lumen (9, 10).

In addition to Atg proteins, phosphatidylinositols (PtdIns) are indispensable for autophagy. Phosphatidylinositol 3-phosphate (PtdIns3P) is essential for PAS organization: the class III phosphatidylinositol 3-kinase complex I, composed of Vps34–Vps15–Atg6–Atg14–Atg38, synthesizes PtdIns3P, which in turn recruits the Atg2–Atg18 complex to supply lipids to the expanding IM (11, 12, 13, 14, 15).

Accumulating evidence indicates that phosphatidylinositol 4-phosphate (PtdIns4P) is also critical for autophagy (16, 17, 18). PtdIns4P primarily accumulates on the Golgi apparatus and the plasma membrane (PM) (19). In *S. cerevisiae*, eight enzymes regulate PtdIns4P metabolism: PtdIns 4-kinases, Pik1 and Stt4; PtdIns4P phosphatases, Sac1, and the dual-specificity Sjl2/Sjl3 (also acting on PtdIns5P); PtdIns4P 5-kinase, Mss4; and PtdIns(4,5)P2 5-phosphatases, Sjl1 and Inp54 (20). Among them, Pik1, Stt4, and Sac1 are associated with macroautophagy, whereas Mss4 is involved in mitochondrial autophagy (16, 18). Pik1 and Stt4 function in distinct subcellular compartments without redundancy: Pik1 localizes to the Golgi apparatus and nucleus, synthesizing PtdIns4P essential for secretory vesicle formation and Golgi maturation (21, 22, 23), whereas Stt4 localizes to the PM and generates PtdIns4P required for actin cytoskeleton organization, cell wall integrity, and sphingolipid homeostasis (23, 24). The PtdIns4P produced by Stt4 serves as a substrate for Mss4, which converts it to PtdIns(4,5)P2 at the PM, thereby regulating endocytosis and actin dynamics (23, 24). Genetic evidence confirms that Pik1 and Stt4 are noninterchangeable; loss of either kinase cannot be compensated by the other, and both are essential for viability (21, 22, 23). Temperature-sensitive (TS) mutants of these genes have thus been widely employed to study their individual functions (16).

A previous study proposed that Golgi-localized Pik1 is required for autophagy and for Atg9 exit from the Golgi in yeast (16). Atg9 is the core transmembrane Atg protein that undergoes Golgi sorting and cycles between the PAS and peripheral sites (25); Atg23 and Atg27 function as Atg9 trafficking factors (26). Atg9 vesicles are required for PAS organization and IM expansion (27). Prior studies have further suggested that Atg9 vesicles act as seeds that establish membrane contact sites to initiate lipid transfer from compartments such as the endoplasmic reticulum (28). Interestingly, the PM-localized Stt4 is also required for autophagy (16). Moreover, the PtdIns4P phosphatase activity of Sac1 has been shown to be important for autophagy (18). Deletion of *SAC1* causes abnormal PtdIns4P accumulation in APs and disrupts recruitment of soluble *N*-ethylmaleimide-sensitive factor attachment protein receptor proteins (18), suggesting a role for Sac1 in later stages, such as fusion.

In mammalian cells, PtdIns4P metabolism has been reported to play roles in autophagy at specific steps. During starvation, ATG9A-positive vesicles deliver PtdIns 4-K IIIβ to AP initiation sites, and PtdIns 4-K IIIβ interacts with ATG9A and ATG13 to control PtdIns4P production at the initiation membrane site (29). In addition, perturbing SAC1 trafficking or activity reshapes cellular PtdIns4P pools and affects autophagy progression (30), and SAC1-mediated restriction of PtdIns4P has been reported to be required for specific AP–lysosome fusion (18). These mammalian studies provide context for examining how spatially defined PtdIns4P pools regulate early autophagy organization in yeast.

Unbiased reporters such as the Osh2 Pleckstrin homology domain have revealed multiple PtdIns4P pools, each dependent on distinct kinases in yeast (31). To clarify their spatial distribution, a high-resolution quick-freeze fracture replica labeling (FRL) EM labeling technique was used. This approach minimizes artifacts by physically immobilizing membrane molecules in their native state and demonstrates that PtdIns4P specifically localizes to the cytoplasmic leaflet of the inner and outer membranes of APs (17). However, the precise functions and mechanisms of PtdIns4P in yeast autophagy remain incompletely understood.

Our study demonstrates that Pik1-generated PtdIns4P is critically required during PAS organization and IM expansion and identifies a mechanism involving its enrichment on Atg9 vesicles.

## Results

### Atg9, Atg17, Atg1, and Atg13 recruitment to the PAS is not affected under Pik1-deficient conditions

**Figure 1:**
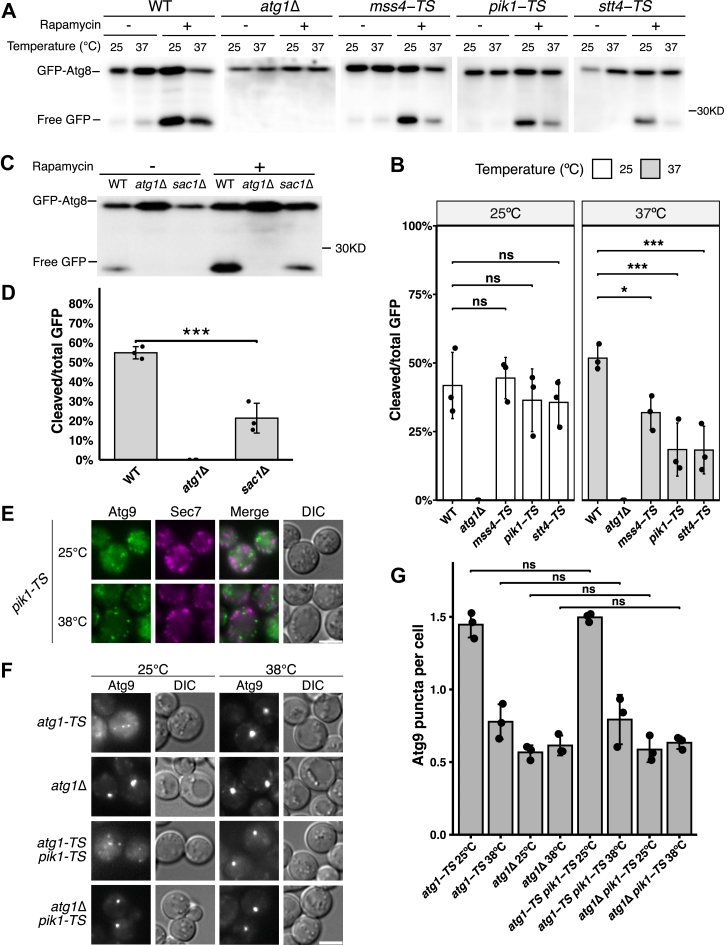
**Recruitment of Atg9 to the PAS is not affected in *pik1* mutant cells.***A*, GFP-Atg8 cleavage assay in WT, *atg1*Δ, *mss4-TS*, *pik1-TS*, and *stt4-TS* cells preincubated at permissive (25 °C) or restrictive (37 °C) temperatures for 30 min, followed by rapamycin treatment for 2 h. Lysates were immunoblotted with anti-GFP antibody. *B*, cleavage ratios in *A* (data are shown as mean ± SD from three independent experiments; each *dot* represents one independent experiment; two-way ANOVA with genotype and temperature as factors, followed by Tukey’s HSD test: ∗*p*< 0.05, ∗∗∗*p*< 0.001, ns *p*≥ 0.05). *C*, GFP-Atg8 cleavage assay in WT, *atg1*Δ, and *sac1*Δ cells treated with rapamycin for 2 h. *D*, cleavage ratios in *C* (data are shown as mean ± SD from three independent experiments; each *dot* represents one independent experiment; one-way ANOVA followed by Tukey’s HSD test: ∗∗∗*p*< 0.001). *E*, exit of Atg9-2×mNeonGreen from the TGN (marked by Sec7-3×mRuby3). *F*, Transport-of-Atg9-after-knocking-out-*ATG1* assay in *atg1-TS*, *atg1*Δ, *atg1-TS pik1-TS*, and *atg1*Δ *pik1-TS* cells. Cells were preincubated at 25 °C or 38 °C (30 min), followed by rapamycin treatment (1 h) (for *E* and *F*). *G*, Atg9-2×mNeonGreen puncta per cell for the strains shown in *F* (data are shown as mean ± SD from three independent experiments; each *dot* represents one independent experiment; n > 50 cells per group; one-way ANOVA followed by Tukey’s HSD test: ns *p*≥ 0.05). Exact *p* values for all statistical comparisons are listed in Table S1. The scale bars represent 4 μm (all images). Atg9, autophagy-related 9; HSD, honestly significant difference; ns, not significant; PAS, preautophagosomal structure; TAKA, transport-of-Atg9-after-knocking-out-ATG1; TGN, trans-Golgi network.

To verify whether the previously reported results for mutant cells involved in PtdIns4P metabolism could be reproduced (16, 17), we used a GFP-Atg8 cleavage assay to assess autophagic activity. In this assay, GFP-Atg8 is transported to the vacuole through autophagy and subsequently degraded by vacuolar proteases, releasing free GFP that serves as an indicator of autophagic activity. WT, *atg1*Δ, and TS mutant cells (*mss4-TS*, *pik1-TS*, and *stt4-TS*) expressing GFP-Atg8 were preincubated at permissive (25 °C) or restrictive (37 °C) temperatures for 30 min, followed by rapamycin treatment (2 h) to induce autophagy. Under rapamycin treatment at 25 °C, WT cells exhibited autophagic activity, whereas *atg1*Δ cells did not (Fig. 1*A*). The *mss4-TS*, *pik1-TS*, and *stt4-TS* mutants displayed autophagic activity comparable to that of WT cells (Fig. 1, *A* and *B*). However, at 37 °C, all three TS mutants showed a significant decrease in autophagic activity relative to WT (Fig. 1, *A* and *B*). In addition, *sac1*Δ cells showed a significant decrease in autophagic activity compared with WT (Fig. 1, *C* and *D*). These results indicate that the PtdIns 4-kinases (Pik1, Stt4), PtdIns4P phosphatase (Sac1), and PtdIns4P 5-kinase (Mss4) are critical for autophagy.

Because Pik1 is the Golgi-localized PtdIns 4-kinase whose dysfunction impairs autophagic activity (Fig. 1*A*) and has been implicated in Atg9 vesicle dynamics in previous work (16), we next tested whether Pik1 deficiency causes a defect in Atg9 exit from the Golgi. Atg9-2×mNeonGreen showed little colocalization with Sec7-3×mRuby3, a trans-Golgi network (TGN) marker, at either 25 °C or 38 °C in *pik1-TS* cells (Fig. 1*E*), suggesting normal exit of Atg9 from the TGN.

In yeast, Atg9 cycles between a cytoplasmic pool and the PAS during autophagy (25, 27). To test whether Atg9 is targeted to the PAS after exiting the TGN, we used the transport-of-Atg9-after-knocking-out-*ATG1* assay, in which Atg1 inactivation blocks recycling of Atg9 from the PAS and causes Atg9 to accumulate at the PAS (25, 32).

We quantified Atg9-2×mNeonGreen puncta per cell in *atg1-TS*, *atg1*Δ, *atg1-TS pik1-TS*, and *atg1*Δ *pik1-TS* cells after preincubation at 25 °C or 38 °C followed by rapamycin treatment. The number of Atg9-2×mNeonGreen puncta per cell was not significantly different between *atg1-TS* and *atg1-TS pik1-TS* cells at either temperature and was also not significantly different between *atg1*Δ and *atg1*Δ *pik1-TS* cells (Fig. 1, *F* and *G*), suggesting that Pik1 deficiency does not markedly affect Atg9 targeting to the PAS.

**Figure 2:**
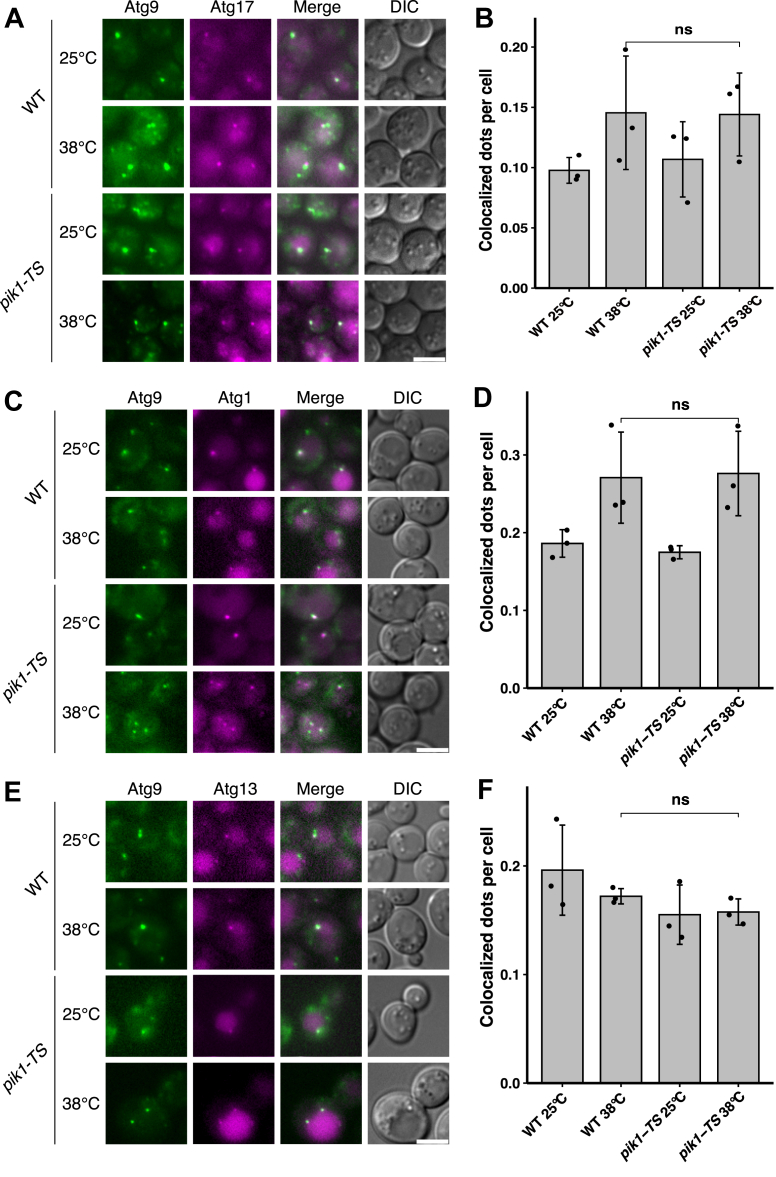
**Recruitment of Atg17, Atg1, and Atg13 to the PAS is not affected in *pik1* mutant cells.***A* and *B*, colocalization of Atg17-3×mRuby3 with Atg9-2×mNeonGreen in WT and *pik1-TS* cells. *C* and *D*, colocalization of Atg1-3×mRuby3 with Atg9-2×mNeonGreen. *E* and *F*, colocalization of Atg13-3×mRuby3 with Atg9-2×mNeonGreen. Cells were preincubated at 25 °C or 38 °C for 30 min, followed by rapamycin treatment for 1 h. *B*, *D*, and *F*, colocalized dots per cell (data are shown as mean ± SD from three independent experiments; each *dot* represents one independent experiment; n > 50 cells per group; one-way ANOVA followed by Tukey’s HSD test: ns *p*≥ 0.05). Exact *p* values for all statistical comparisons are listed in Table S1. The scale bars represent 4 μm (all images). Atg, autophagy-related; HSD, honestly significant difference; ns, not significant; PAS, preautophagosomal structure.

We next examined the recruitment of other early scaffolding proteins to the PAS in *pik1-TS* cells at permissive and restrictive temperatures. Atg9-2×mNeonGreen and Atg17-3×mRuby3 colocalized in WT and *pik1-TS* cells at 25 °C and 38 °C (Fig. 2*A*). Quantitative analysis revealed no significant difference in the proportion of Atg9–Atg17 colocalized dots per cell between WT and *pik1-TS* cells at 38 °C (Fig. 2*B*). Moreover, the number of Atg1–Atg9 and Atg13–Atg9 colocalized dots per cell at 38 °C was not significantly different between *pik1-TS* and WT cells (Fig. 2, *C*–*F*). Collectively, these findings demonstrate that even under Pik1-deficient conditions, Atg9 exits normally from the Golgi and is subsequently recruited to the PAS, where it colocalizes with Atg17, Atg1, and Atg13.

### Downstream Atg protein recruitment is impaired under Pik1-deficient conditions

To examine the effect of Pik1-generated PtdIns4P on AP biosynthesis, we analyzed the recruitment of downstream Atg proteins to the PAS in *pik1-TS* mutant cells using fluorescence microscopy. Cells were preincubated at the respective temperatures for 30 min and treated with rapamycin.

**Figure 3:**
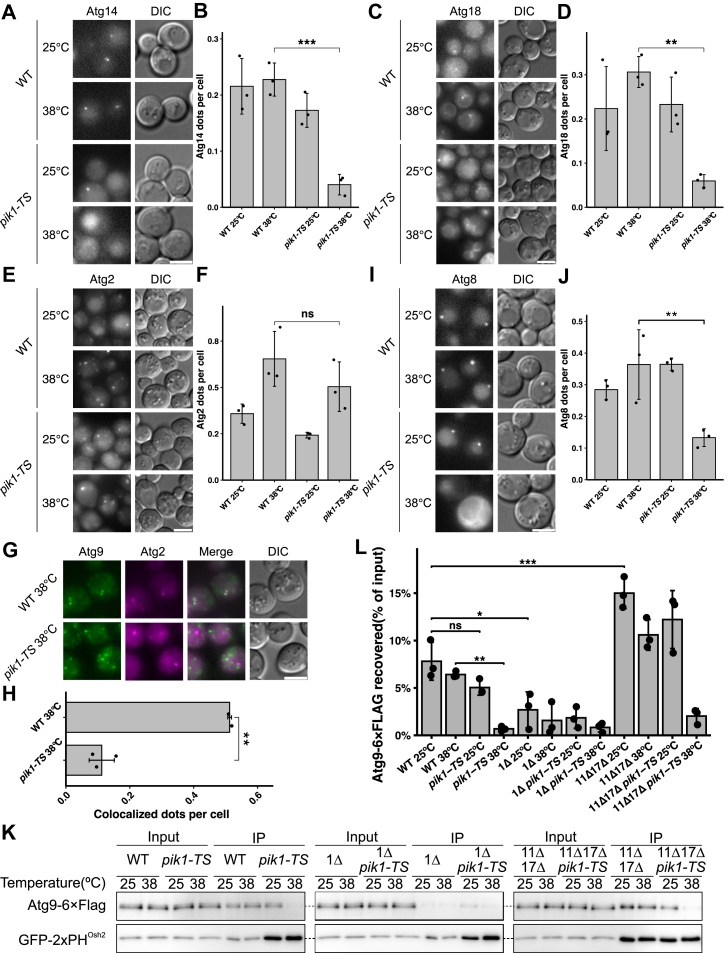
**Impaired recruitment of Atg14, Atg18, Atg2, and Atg8 in *pik1* mutant cells.***A* and *B*, localization of Atg14-3×mRuby3 in WT and *pik1-TS* at 25 °C or 38 °C. *C* and *D*, localization of Atg18-3×mRuby3. *E* and *F*, localization of Atg2-3×mRuby3. *B*, *D*, and *F*, Atg14, Atg18, and Atg2 dots per cell (data are shown as mean ± SD from three independent experiments; each *dot* represents one independent experiment; n > 50 cells per group; one-way ANOVA followed by Tukey’s HSD test: ∗∗*p*< 0.01, ∗∗∗*p*< 0.001, ns *p*≥ 0.05). *G*, colocalization of Atg2-3×mRuby3 with Atg9-2×mNeonGreen. *H*, Atg2 and Atg9 colocalized dots per cell at 38 °C (data are shown as mean ± SD from three independent experiments; each *dot* represents one independent experiment; unpaired, two-tailed Student’s *t* test: ∗∗*p*< 0.01). *I*, localization of mRuby3-Atg8. *J*, Atg8 dots per cell (data are shown as mean ± SD from three independent experiments; each *dot* represents one independent experiment; n > 50 cells per group; one-way ANOVA followed by Tukey’s HSD test: ∗∗*p*< 0.01). The scale bars represent 4 μm (all images). *K*, coimmunoprecipitation of Atg9-6×FLAG with GFP-2×PH^Osh2^ in the indicated genotypes. Immunoblots were probed with anti-FLAG (*top*) and anti-GFP (*bottom*) antibodies. For immunoprecipitation (IP)/input calculations, band intensities were corrected for the 7.5-fold concentration of the IP eluate relative to the input. *L*, quantification of Atg9-6×FLAG recovered in the GFP-2×PH^Osh2^ immunoprecipitate, expressed as corrected IP/input ratios (as in *K*), in WT, *pik1-TS*, *atg1*Δ, *atg11*Δ, *atg17*Δ, and the indicated combinations (data are shown as mean ± SD from three independent experiments; each *dot* represents one independent experiment; one-way ANOVA followed by Tukey’s HSD test: ∗*p*< 0.05, ∗∗*p*< 0.01, ∗∗∗*p*< 0.001, ns *p*≥ 0.05). Exact *p* values for all statistical comparisons are listed in Table S1. Cells were preincubated at 25 °C or 38 °C (30 min), followed by rapamycin treatment (1 h). Atg, autophagy-related; HSD, honestly significant difference; ns, not significant.

At 25 °C, Atg14-3×mRuby3 formed distinct dots in WT and *pik1-TS* cells (Fig. 3*A*). However, at 38 °C, the number of Atg14-3×mRuby3 dots in *pik1-TS* cells was markedly reduced compared with that of WT (Fig. 3, *A* and *B*), indicating that PAS recruitment of Atg14 is impaired under Pik1-deficient conditions. Similarly, the number of Atg18-3×mRuby3 dots was significantly lower in *pik1-TS* cells than in WT cells at 38 °C (Fig. 3, *C* and *D*), suggesting that PAS recruitment of Atg18 is likewise impaired under Pik1-deficient conditions.

Although the number of Atg2-3×mRuby3 dots per cell did not differ significantly between WT and *pik1-TS* cells at 38 °C (Fig. 3, *E* and *F*), fluorescence microscopy revealed a marked defect in the colocalization between Atg2 and Atg9. At 38 °C, the proportion of Atg2-3×mRuby3 that colocalized with Atg9-2×mNeonGreen was significantly reduced in *pik1-TS* cells (Fig. 3, *G* and *H*), indicating that PAS recruitment of Atg2 is impaired when Pik1 activity is lost. Consistent with a defect in PAS formation, Atg8 dot formation was also reduced in *pik1-TS* cells at 38 °C (Fig. 3, *I* and *J*).

Taken together, these results suggest that Pik1-generated PtdIns4P is required for the recruitment of downstream Atg proteins, including the PtdIns 3-kinase complex I (containing Atg14), the Atg2–Atg18 complex, and Atg8 (Fig. 3, *A*–*J*).

### PtdIns4P enrichment on Atg9 vesicles requires Pik1 activity

Under Pik1-deficient conditions, Atg9 exits from the Golgi and is recruited to the PAS (Figs. 1, *E*–*G*, and 2, *A*–*F*). Under these conditions, the recruitment of other Atg proteins is impaired (Fig. 3, *A*–*J*), suggesting that the recruitment of downstream Atg proteins depends on Golgi-derived PtdIns4P. We therefore hypothesized that Pik1-generated PtdIns4P is enriched on Atg9 vesicles. To test this, we immunoprecipitated PtdIns4P-containing membranes using GFP-2×PH^Osh2^, a PtdIns4P-specific probe, and examined whether Atg9 was coimmunoprecipitated. Cells expressing GFP-2×PH^Osh2^ and Atg9-6×FLAG were preincubated at the respective temperatures for 30 min, followed by rapamycin treatment.

At 25 °C and 38 °C, Atg9-6×FLAG efficiently coimmunoprecipitated with GFP-2×PH^Osh2^ in WT cells (Fig. 3*K*), showing that Atg9 is coimmunoprecipitated with GFP-2×PH^Osh2^. In *pik1-TS* cells, Atg9-6×FLAG also coimmunoprecipitated efficiently with GFP-2×PH^Osh2^ at 25 °C; however, precipitated Atg9-6×FLAG was significantly reduced at 38 °C (Fig. 3, *K* and *L*). We examined the precipitates by fluorescence microscopy and found that Atg9-3×mRuby was associated with GFP-2×PH^Osh2^ in WT cell lysates (Fig. S1, *A* and *B*). This association was abolished by the treatment with 1% Triton X-100 (Fig. S1, *A* and *B*). These results suggest that Atg9 is coimmunoprecipitated with membranes containing PtdIns4P that is generated by Pik1.

As mentioned above, Atg9 cycles between a cytoplasmic pool and the PAS (25, 27). In *atg1*Δ cells, Atg9 accumulates at the PAS (Fig. 1, *F* and *G*), whereas in *atg11*Δ *atg17*Δ cells, Atg9 is present as mobile cytoplasmic vesicles because of the absence of the PAS (27). In *atg1*Δ cells, precipitated Atg9-6×FLAG was significantly reduced compared with WT cells (Fig. 3, *K* and *L*). In *atg11*Δ *atg17*Δ cells, precipitated Atg9-6×FLAG was significantly increased compared with WT cells (Fig. 3, *K* and *L*). These results suggest that cytoplasmic Atg9 vesicles are preferentially immunoprecipitated with GFP-2×PH^Osh2^ compared with PAS-targeted Atg9 vesicles.

Together, these results suggest that PtdIns4P is enriched on Atg9 vesicles depending on Pik1 activity.

### PtdIns4-kinases are necessary for IM expansion

Next, we investigated the role of PtdIns4P in IM expansion. To do so, we analyzed the recruitment of mRuby3-Atg8 to the PAS. Cells expressing mRuby3-Atg8 were preincubated at the respective temperatures for 30 min, followed by rapamycin treatment. The number of mRuby3-Atg8 dots per cell was significantly reduced in *pik1-TS* cells relative to WT cells at 38 °C (Fig. 3, *I* and *J*). To improve the visualization of Atg proteins, we employed the prApe1-overexpression system, which allows clearer tracking because of the lower mobility of the large Ape1 droplet. Cells expressing mNeonGreen-Atg8 and overexpressing prApe1 were preincubated at the respective temperatures for 30 min and then treated with rapamycin.

**Figure 4:**
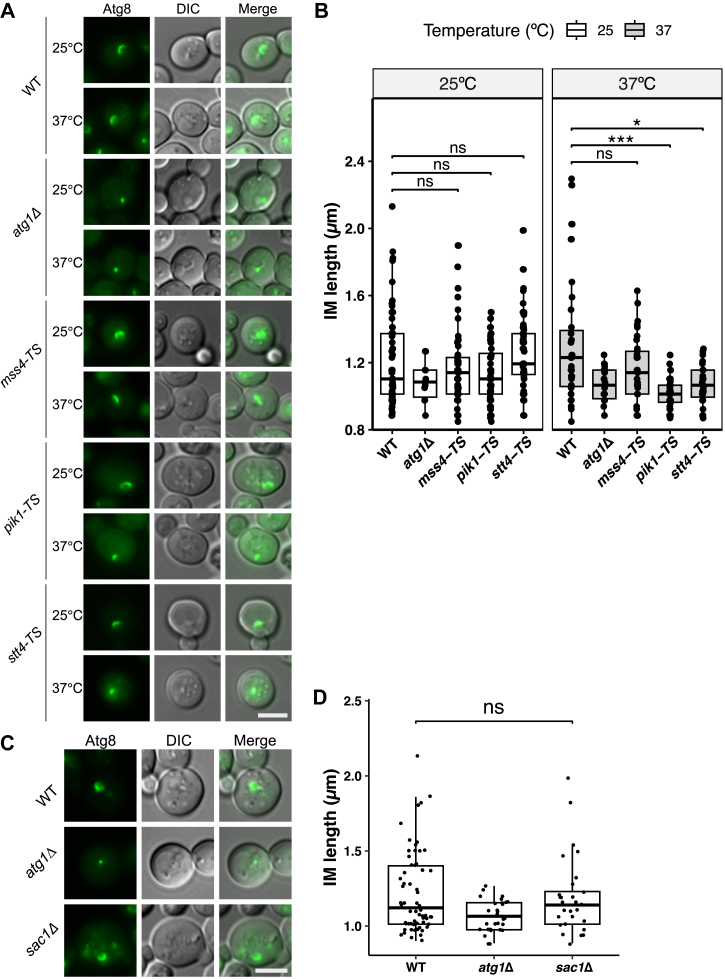
**PtdIns4-kinases are required for IM expansion.***A*, representative images of IMs in WT, *atg1*Δ, *mss4-TS*, *pik1-TS*, and *stt4-TS* cells. Cells expressing mNeonGreen-Atg8 and overexpressing prApe1 (from plasmid pYEX-BX[prApe1]) were preincubated at 25 °C or 37 °C (30 min), treated with rapamycin (1 h), and imaged. *B*, quantification of IM lengths (boxplots and quartiles): WT (n = 96), *atg1*Δ (32), *mss4-TS* (85), *pik1-TS* (98), *stt4-TS* (78) (Wilcoxon's test: ∗*p*< 0.05, ∗∗∗*p*< 0.001, ns *p*≥ 0.05). *C*, IM length in WT, *atg1*Δ, and *sac1*Δ cells. Cells were treated with rapamycin (1 h) at 30 °C. *D*, quantification of IM lengths: WT (n = 62), *atg1*Δ (31), and *sac1*Δ (29) (Wilcoxon's test: ns *p*≥ 0.05). Exact *p* values for all statistical comparisons are listed in Table S1. The scale bars represent 4 μm (all images). IM, isolation membrane; ns, not significant; PtdIns, phosphatidylinositol.

At 25 °C, WT and TS mutant cells displayed cup-shaped IMs, whereas *atg1*Δ cells showed dot-shaped structures (Fig. 4*A*). At 37 °C, WT and *mss4-TS* cells exhibited elongated IMs, but *pik1-TS* and *stt4-TS* cells showed shorter rod-like structures (Fig. 4*A*). Quantitative analysis confirmed that at 37 °C, IM length was significantly lesser in *pik1-TS* and *stt4-TS* cells compared with WT, whereas the length of *mss4-TS* cells did not differ significantly from that of the WT (Fig. 4*B*). These results indicate that Pik1 and Stt4, but not Mss4, are required for IM expansion.

To assess whether Sac1 is necessary for IM expansion, *sac1*Δ and WT cells expressing mNeonGreen-Atg8 and overexpressing prApe1 were treated with rapamycin. *sac1*Δ and WT cells exhibited cup-shaped IMs (Fig. 4*C*). Quantification showed no significant difference in IM length between *sac1*Δ and WT cells (Fig. 4*D*), indicating that Sac1 is dispensable for IM expansion. Interestingly, although *sac1*Δ cells are defective in autophagy (Fig. 1, *C* and *D*), their IM expansion was not impaired, suggesting that Sac1 may function at later stages of autophagy after IM expansion has occurred.

**Figure 5:**
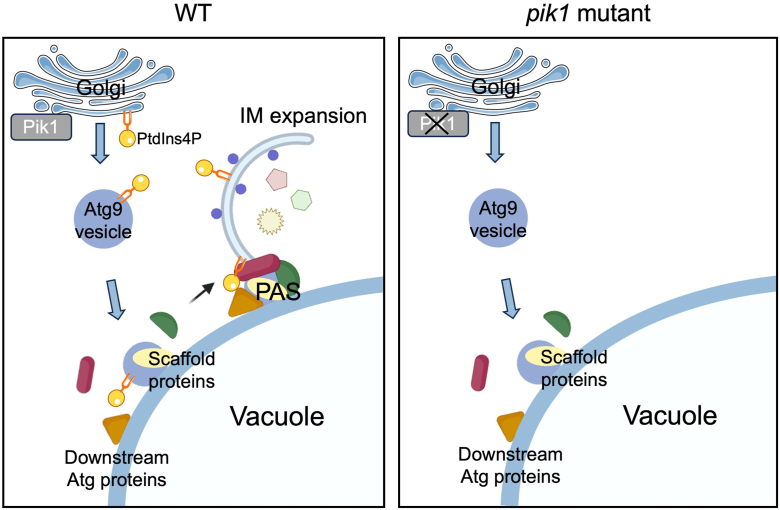
**Model of Golgi-derived PtdIns4P function.** WT (*left*), under normal conditions, PtdIns4P enrichment on Golgi-derived Atg9 vesicles enables the recruitment of the PtdIns 3-kinase complex I to the PAS. *pik1* mutant (*right*), under Pik1-deficient conditions, PAS scaffold proteins (Atg9/Atg17/Atg1/Atg13) remain recruited, but the absence of Golgi-derived PtdIns4P impairs the recruitment of downstream Atg proteins (including the PtdIns 3-kinase complex I, the Atg2–Atg18 complex, and Atg8) and inhibits IM expansion. Image adapted from Servier Medical Art (https://smart.servier.com/), as licensed under CC BY 4.0 (https://creativecommons.org/licenses/by/4.0/). Atg, autophagy-related; IM, isolation membrane; PAS, preautophagosomal structure; PtdIns 3, phosphatidylinositol 3; PtdIns4P, phosphatidylinositol 4-phosphate.

In this study, we demonstrate that Pik1 activity is essential for the enrichment of PtdIns4P on Atg9 vesicles during autophagy. Following the recruitment of Atg9 vesicles to the PAS, PtdIns4P present on these vesicles is required for the recruitment of downstream Atg proteins, including the PtdIns 3-kinase complex I, the Atg2–Atg18 complex, and Atg8. Incomplete PAS organization may underlie the IM expansion defects observed in *pik1*-deficient cells (Fig. 5).

## Discussion

This study establishes that Golgi-derived PtdIns4P, synthesized by Pik1, is essential for PAS organization as Pik1 activity promotes the enrichment of PtdIns4P on Atg9 vesicles and enables the subsequent recruitment of downstream Atg proteins.

### Recruitment of Atg9 vesicles to the PAS in Pik1-defective cells

Consistent with the findings of a previous study (16), the GFP-Atg8 cleavage assay showed that autophagic activity is reduced in *pik1-TS* cells (Fig. 1, *A* and *B*). At the restrictive temperature, we observed no notable colocalization between Sec7, a TGN marker, and Atg9 during autophagy (Fig. 1*E*), indicating that Atg9 vesicles properly exit from the TGN. Although a prior report described colocalization between Atg9 and Sec7 under restrictive conditions (16), differences in experimental parameters and strain backgrounds (BY4742 *versus* W303) may account for this discrepancy. Under our study conditions, Atg9 leaves the TGN and is normally targeted to the PAS, as supported by the results of the transport-of-Atg9-after-knocking-out-*ATG1* assay and the observed colocalization of Atg17/Atg1/Atg13 and Atg9 in *pik1* mutant cells (Fig. 2).

### Pik1 activity promotes enrichment of PtdIns4P on cytoplasmic Atg9 vesicles

Atg9 was coimmunoprecipitated with GFP-2×PH^Osh2^ in *pik1-TS* cells at the permissive temperature but not at the restrictive temperature (Fig. 3, *K* and *L*). This suggests that Pik1 is required for the enrichment of PtdIns4P on cytoplasmic Atg9 vesicles. Atg9 was hardly coimmunoprecipitated in the absence of *ATG1*, whereas it was efficiently coimmunoprecipitated in the absence of *ATG11* and *ATG17* (Fig. 3, *K* and *L*), suggesting that cytoplasmic Atg9 vesicles are preferentially recovered compared with PAS-targeted Atg9 vesicles. Because this result is indirect, further verification through more direct approaches, such as visualizing PtdIns4P on isolated Atg9 vesicles by immunoelectron microscopy, is necessary to strengthen this conclusion. A previous study reported that PtdIns4P localizes to the IM based on immunoelectron microscopy (17), suggesting that PtdIns4P detected on the IM is, at least in part, derived from Atg9 vesicles that act as seeds for IM formation.

### Golgi-derived PtdIns4P is involved in the recruitment of downstream Atg proteins

In *pik1*-*TS* cells, PAS localization of the PtdIns 3-kinase complex I, labeled with Atg14, is impaired (Fig. 3, *A* and *B*). As PtdIns3P generation at the PAS is required for the recruitment of downstream Atg proteins (12), the recruitment of the Atg2–Atg18 complex and Atg8 is also impaired (Fig. 3, *C*–*J*). These findings suggest that PtdIns4P influences the PAS targeting of the PtdIns 3-kinase complex I. We propose three possible mechanisms: first, the PtdIns 3-kinase complex I may directly bind to PtdIns4P on Atg9 vesicles; second, PtdIns4P may interact with Atg9 to form a PtdIns4P–Atg9 complex that facilitates the association of the PtdIns 3-kinase complex I with Atg9 vesicles; and third, the physicochemical properties conferred by the presence of PtdIns4P may enhance the interaction between the PtdIns 3-kinase complex I and Atg9 vesicles.

### PtdIns4P-binding proteins in autophagy

In this study, we show that PtdIns4P contributes to the recruitment of the PtdIns 3-kinase complex I (Fig. 3, *A* and *B*). A previous study using the SDS-digested freeze-FRL technique demonstrated that PtdIns4P signals are detected on both leaflets of membranes comprising the IM under EM (17). SDS-FRL involves rapid freezing, freeze fracture, and platinum/carbon replication, followed by SDS treatment to remove membranes and membrane surface–binding proteins. This exposed phospholipid headgroups for antibody labeling (17, 33). We examined colocalization of GFP-2×PH^Osh2^, a PtdIns4P probe, with the IM labeled by mRuby3-Atg8 under a fluorescence microscope and found that the IM was not labeled by GFP-2×PH^Osh2^ (Fig. S3, *A* and *B*). One possible explanation for this discrepancy is that, under *in vivo* conditions, nearly all PtdIns4P on the IM is occupied by its effector proteins, leaving little PtdIns4P accessible to GFP-2×PH^Osh2^. This finding suggests the presence of PtdIns4P-binding proteins on the IM. Further screening and characterization of such PtdIns4P-binding proteins will help elucidate the functional roles of PtdIns4P on the IM.

### IM expansion requires both spatially distinct PtdIns4P pools

Previous studies have identified two distinct PtdIns4P pools in yeast: a Golgi pool generated by Pik1 and a PM pool generated by Stt4 (22, 31, 34). In this study, we found that *pik1-TS* cells exhibited defects in IM expansion (Fig. 4, *A* and *B*), and a similar phenotype was observed in *stt4*-*TS* cells (Fig. 4, *A* and *B*). These results indicate that both PtdIns4P pools are essential for efficient IM expansion. Based on our findings, the mechanism by which Pik1-generated PtdIns4P contributes to IM expansion likely involves promoting the recruitment of downstream Atg proteins. In contrast, the mechanism through which Stt4-generated PtdIns4P at the PM supports IM expansion remains unclear. Given the distinct PtdIns4P distribution patterns observed in *stt4* and *pik1* mutant cells (31), we propose that these two kinases contribute to autophagy through different, nonredundant mechanisms. The precise role of Stt4 in this process, however, remains to be clarified.

While this study establishes the essential role of Golgi-derived PtdIns4P in PAS organization and downstream Atg protein recruitment, the mechanisms underlying PtdIns4P-mediated recruitment are still not fully understood. Future work should employ structural and lipid-binding analyses to identify specific PtdIns4P effectors involved in autophagy. Furthermore, the distinct contribution of Stt4-generated PtdIns4P at the PM to AP biogenesis warrants detailed investigation as it may function *via* a separate pathway. The distinct yet complementary roles of PtdIns4P and PtdIns3P add further complexity to AP formation, suggesting that their spatiotemporal coordination fine-tunes autophagy. Elucidating the mechanisms governing the interconversion and regulation of these phosphoinositides may be critical to develop a comprehensive understanding of AP biogenesis.

## Experimental procedures

### Strains and plasmids

Yeast strains and plasmids used in this study are listed in Tables 1 and 2. Cells were cultured in YPD medium (1% w/v Bacto yeast extract, 2% w/v Bacto peptone, and 2% w/v glucose). *Escherichia coli* cells harboring plasmids were cultured in LB medium (1% w/v Bacto tryptone, 0.5% w/v Bacto yeast extract, and 1% w/v NaCl) supplemented with ampicillin at a concentration of 100 μg/ml. For TS mutants, cells were grown at 25 °C to midlog phase and then transferred to 37 °C or 38 °C for 30 min to inactivate mutant proteins. Unless otherwise indicated, all cultures were maintained at 30 °C. To activate the Cu^2+^-inducible *CUP1* promoter, cells were grown for 1 day in a medium containing 250 μM CuSO_4_ prior to experiments. Autophagy was induced by adding 400 ng/ml rapamycin.

**Table 1:** Yeast strains used in this study

Strain	Genotype	Source
BY4741	*MAT***a***his3Δ1 leu2Δ0 met15Δ0 ura3Δ0*	Lab stock
*atg1Δ*	BY4741; *atg1Δ::kanMX*	Lab stock
*mss4-TS*	BY4741; *mss4-103::kanMX*	Lab stock
*pik1-TS*	BY4741; *pik1-104::kanMX*	Lab stock
*stt4-TS*	BY4741; *stt4-4::kanMX*	Lab stock
*sac1*Δ	BY4741; *sac1Δ::kanMX*	Lab stock
W303-1A	*ADE2 leu2-3 his3-11, 15 trp1-1 ura3-1 can1-100*	Lab stock
TYS185	W303-1A; *his3Δ::mRuby3-ATG8:HIS3*	Lab stock
LHYC001	W303-1A; *Atg14::3*×*mRuby3-natNT2*	This study
LHYC002	W303-1A; *Atg17::3*×*mRuby3-natNT2Atg9::2*×*mNeonGreen-kanMX*	This study
LHYC003	W303-1A; *Sec7::3*×*mRuby3-natNT2Atg9::2*×*mNeonGreen-kanMX*	This study
LHYC004	W303-1A; *Atg18::3*×*mRuby3-natNT2*	This study
LHYC005	W303-1A; *Atg2::3*×*mRuby3-natNT2Atg9::2*×*mNeonGreen-kanMX*	This study
LHYC006	W303-1A; *pik1-104::kanMX*	This study
LHYC007	LHYC006; *his3Δ::mRuby3-ATG8:HIS3*	This study
LHYC008	LHYC006; *Atg14::3*×*mRuby3-natNT2*	This study
LHYC009	LHYC006; *Atg17::3*×*mRuby3-natNT2Atg9::2*×*mNeonGreen-kanMX*	This study
LHYC010	LHYC006; *Sec7::3*×*mRuby3-natNT2Atg9::2*×*mNeonGreen-kanMX*	This study
LHYC011	LHYC006; *Atg18::3*×*mRuby3-natNT2*	This study
LHYC012	LHYC006; *Atg2::3*×*mRuby3-natNT2Atg9::2*×*mNeonGreen-kanMX*	This study
LHYC013	W303-1A; *pep4Δ::CgHIS3 Atg9-3*×*EcBAP::kanMX4 leu2Δ::BirA::hphNT1*	This study
LHYC014	LHYC006; *pep4Δ::CgHIS3 Atg9-3*×*EcBAP::kanMX4 leu2Δ::BirA::hphNT1*	This study
LHYC015	W303-1A; *Atg1::3*×*mRuby3-natNT2Atg9::2*×*mNeonGreen-kanMX*	This study
LHYC016	W303-1A; *Atg13::3*×*mRuby3-natNT2Atg9::2*×*mNeonGreen-kanMX*	This study
LHYC017	LHYC006; *Atg1::3*×*mRuby3-natNT2Atg9::2*×*mNeonGreen-kanMX*	This study
LHYC018	LHYC006; *Atg13::3*×*mRuby3-natNT2Atg9::2*×*mNeonGreen-kanMX*	This study
LHYC019	W303-1A; *atg1*Δ*::natNT2Atg9::2*×*mNeonGreen-kanMX*	This study
LHYC020	LHYC006; *atg1*Δ*::natNT2Atg9::2*×*mNeonGreen-kanMX*	This study
LHYC021	LHYC013; *atg1*Δ*::natNT2*	This study
LHYC022	LHYC014; *atg1*Δ*::natNT2*	This study
LHYC023	LHYC013; *atg11*Δ*::natNT2atg17*Δ*::ctTRP1*	This study
LHYC024	LHYC014; *atg11*Δ*::natNT2atg17*Δ*::ctTRP1*	This study
LHYC025	W303-1A; *Atg9::3*×*mRuby3-natNT2*	This study
LHYC026	LHYC006; *Atg9::3*×*mRuby3-natNT2*	This study

Minus Symbol (−)Bold denotes the mating-type allele “a” in Saccharomyces cerevisiae.

**Table 2:** Plasmids used in this study

Name	Description	Marker	Source
pRS316[GFP-Atg8]	Centromeric plasmid for expression of GFP-Atg8 using the *ATG8* promoter	*URA3*	(37)
pFA6a-3×mRuby3-natNT2	PCR template for generation of C-terminally 3×mRuby3-fused proteins		Lab stock
pFA6a-2×mNeonGreen-kanMX	PCR template for generation of C-terminally 2×mNeonGreen-fused proteins		Lab stock
pFA6a-natNT2	PCR template for natNT2 knockout cassette		Lab stock
pFA6a-ctTRP1	PCR template for TRP1 knockout cassette		Lab stock
pHYA1523	Integration plasmid for expression of EGFP-2×PH using the *TDH3* promoter	*URA3*	(38)
pRS315[GFP-2×PH^Osh2^]	Centromeric plasmid for expression of EGFP-2×PH using the *TDH3* promoter	*LEU2*	This study
pRS316[GFP-2×PH^Osh2^]	Centromeric plasmid for expression of EGFP-2×PH from the *TDH3* promoter	*URA3*	This study
pRS316[Atg9-6×FLAG]	Centromeric plasmid for expression of Atg9-6×FLAG using the *ATG9* promoter	*URA3*	(39)
p416[GFP-2×FYVE]	Centromeric plasmid for expression of EGFP-2×FYVE using the *TEF* promoter	*URA3*	(13)
pYEX-BX[prApe1]	2μ plasmid for expression of prApe1 using the *CUP1* promoter	*URA3*	(8)
pRS314[atg1-TS]	Centromeric plasmid for expression of atg1-TS using the *ATG1* promoter	*TRP1*	(37)
pRS314	Empty centromeric vector	*TRP1*	Lab stock

The TS allele *pik1-TS*, which was originally in the BY4741 background (genotype: *MATa his3Δ1 leu2Δ0 met15Δ0 ura3Δ0 pik1-104::KanMX*) was introgressed into the W303 genetic background (*MATα ADE2 leu2-3 his3-11,15 trp1-1 ura3-1 can1-100*). This was done *via* four consecutive rounds of backcrossing and tetrad dissection. Cells expressing C-terminally 2×mNeonGreen- or 3×mRuby3-fused proteins were generated using a PCR-based gene modification method (35).

Plasmid pRS315/6[GFP-2×PH^Osh2^] was constructed by cloning the GFP-2×PH^Osh2^ fragment from pHYA1523 (a gift from Dr Shigeomi Shimizu, Institute of Science Tokyo), digested with SpeI and SalI, into pRS315/6 digested with the same restriction enzymes.

To maintain plasmid expression, yeast transformants were cultured in SD/CA medium (0.17% w/v Difco yeast nitrogen base without amino acids and ammonium sulfate, 0.5% w/v ammonium sulfate, 0.5% w/v Bacto casamino acids, and 2% w/v glucose) or SD/DO medium (0.17% w/v Difco yeast nitrogen base without amino acids and ammonium sulfate, 0.5% w/v ammonium sulfate, 2% w/v glucose), each supplemented with the appropriate amino acids.

### Fluorescence microscopy

Cells were cultured to a concentration of approximately 2 × 10^7^cells/ml. To induce autophagy, transformed cells were treated with 400 ng/ml rapamycin for 1 h. Cells were collected by microcentrifugation, resuspended in the remaining medium, and observed by fluorescence microscopy. An IX83 inverted microscope system (Olympus) equipped with a CoolSNAP HQ CCD camera (Nippon Roper) was used for image acquisition. U-FGFP and U-FRFP filter sets (Olympus) were used for mNeonGreen/GFP and mRuby3 visualization, respectively. Images were acquired using MetaVue software (Molecular Devices).

### Quantification of microscopic images by Qautas

GFP-Atg8-labeled structures were extracted from microscopic images and morphometrically analyzed using ImageJ software (version 1.53q; National Institutes of Health) with Qautas (quantitative autophagy-related structure analysis system), as described previously (36). Fluorescence intensity was first normalized. Microscopic images were filtered with a bandpass filter (2–5 pixels) to detect abrupt changes in intensity, and the resulting images were binarized using the RenyiEntropy algorithm with an automatically determined threshold. Structures were then extracted from the binarized images. Nine parameters, area, major axis length, minor axis length, perimeter, angle, circularity, aspect ratio, roundness, and solidity, were obtained from each structure using the particle analysis function in ImageJ.

As training data, 359 GFP-Atg8-labeled structures were manually classified as either elongated or dot shaped. The training dataset was generated using the IM visualization method, and the discriminator was constructed with the Random Forest algorithm (randomForest version 4.6-10) in R (version 4.1.2). The nine parameters extracted from ImageJ were used as input features for this discriminator. In the Random Forest algorithm, multiple decision trees were built, each generating classification criteria for dot-shaped or elongated structures based on two randomly selected parameters. Half of the manually classified data were used for model training. and the remaining half was used for accuracy testing. The importance of each parameter was calculated based on the accuracy rate of decision trees that used that parameter. The final classification accuracy of the discriminator was 91.1%.

### SDS-PAGE and Western blotting

Cells cultured in SD/CA medium (2 × 10^7^cells/ml) were subjected to alkaline trichloroacetic acid lysis, followed by SDS-PAGE and Western blotting analysis. SDS-PAGE was performed using a 10% w/v acrylamide gel.

Proteins were transferred to polyvinylidene difluoride membranes (Immobilon-P; Millipore) using a semidry transfer apparatus (Bio-Rad) at 2 mA per 1 cm^2^ for 15 min. Following transfer, the membranes were blocked with 2% w/v skim milk in Tris-buffered saline containing 0.05% v/v Tween-20 (TBST) for 30 min at room temperature. Membranes were then incubated with primary anti-GFP antibodies (Clontech, JL-8; 1:5000 dilution) and/or anti-FLAG (Sigma-Aldrich, M2; 1:5000 dilution) for 60 min at room temperature. After three washes with TBST, membranes were incubated with horseradish peroxidase–labeled antimouse secondary antibodies (Promega; 1:5000 dilution) for 30 min, followed by additional TBST washes. Chemiluminescent signals generated using the ImmunoStar LD reagent (FUJIFILM) were detected with the IR-LAS 1000 imaging system (FUJIFILM).

## Immunoprecipitation

For immunoprecipitation of GFP-2×PH^Osh2^, cells expressing GFP-2×PH^Osh2^ from a pRS315 single-copy plasmid and Atg9-6×FLAG from a pRS316 single-copy plasmid were used. Cells cultured in SD/DO medium (2 × 10^7^cells/ml) were harvested and washed twice. Cells were then disrupted in a Multi-Beads Shocker (Yasui Kikai) using 0.5-mm glass beads in HSE buffer (25 mM Hepes–KOH, pH 7.2, 750 mM sorbitol, and 5 mM EDTA) containing 0.5 mg/ml bovine serum albumin and 50 mM NaCl, supplemented with protease inhibitors (Protease Inhibitor Cocktail, Sigma; 1× final) and PMSF (Sigma; 1 mM final). After centrifugation at 20,000*g* for 20 min at 4 °C, the supernatants were incubated with GFP-Trap (Proteintech) for 2 h at 4 °C. The beads were collected using a magnetic stand and washed three times with HSE buffer containing 0.5 mg/ml bovine serum albumin and 250 mM NaCl. For detergent control, 1% (v/v) Triton X-100 was included in the wash buffer during the bead-washing steps. After final washing with HSE buffer, bound proteins were eluted with SDS-PAGE sample buffer.

## Data availability

All data reported in this study are available from the corresponding author upon reasonable request. This article does not include any original code. Additional information necessary to reanalyze the data presented here can also be obtained from the corresponding author upon request.

## Supporting information

This article contains supporting information.

## Conflict of interest

The authors declare that they have no conflicts of interest with the contents of this article.
