# Supplementary material for: Golgi-derived phosphatidylinositol 4-phosphate is crucial for organization of the preautophagosomal structure
Source: J Biol Chem. 2026 Apr 9;302(6):111445. doi: 10.1016/j.jbc.2026.111445 (PMC13156747; doi:10.1016/j.jbc.2026.111445)
Supplement: Supporting Information [file mmc2.pdf]

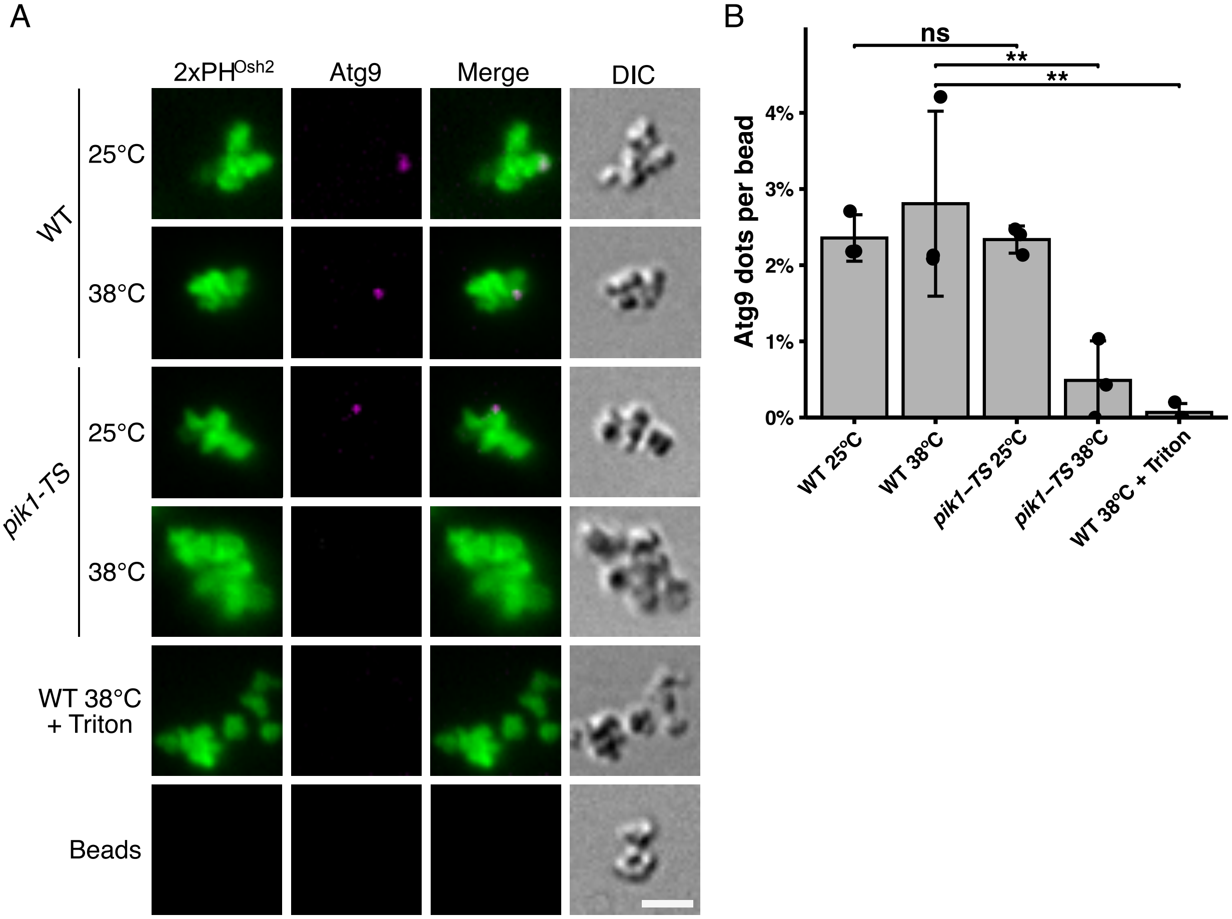

Fig. S1. Fluorescence microscopy of GFP-Trap beads after immunoprecipitation of GFP-2×PH^{Osh2} from cell lysates

A. Representative fluorescence images of GFP-Trap beads after immunoprecipitation of GFP-2×PH^{Osh2} (green) from WT and *pik1-TS* cells expressing Atg9-3×mRuby (magenta) at 25°C or 38°C. A detergent control (WT 38°C + Triton X-100) and a beads-only control processed in parallel (no lysate) are shown. B. Quantification of Atg9-3×mRuby dots per bead for the conditions in A (data are shown as mean ± SD from three independent experiments; each dot represents one independent experiment; n > 100 beads per group; one-way ANOVA followed by Tukey's HSD test: ** $P < 0.01$, ns $P \geq 0.05$). Exact P values for all statistical comparisons are listed in Table S1. Cells were pre-incubated at 25°C or 38°C (30 min), followed by rapamycin treatment for 1 hr. Scale bars: 2 μm.

Fig. S2

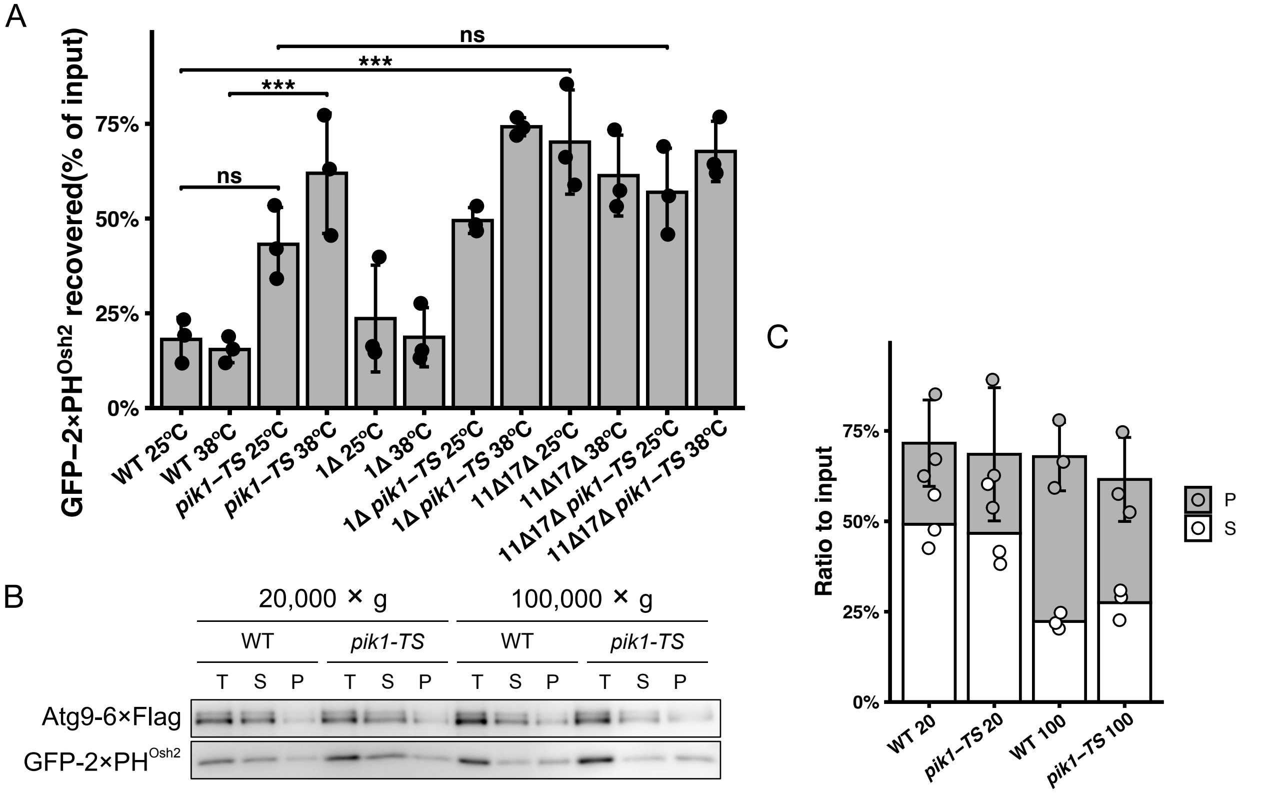

Fig. S2. Distribution of GFP-2×PH^{Osh2} in WT and *pik1-TS* lysates

A. Quantification of GFP-2×PH^{Osh2} recovered in the immunoprecipitate in the experiment shown in Fig. 3K, expressed as corrected IP/Input ratios (data are shown as mean ± SD from three independent experiments; each dot represents one independent experiment; one-way ANOVA followed by Tukey's HSD test: *** $P < 0.001$, ns $P \geq 0.05$). Exact P values for all statistical comparisons are listed in Table S1. B. Differential centrifugation of cell lysates from cells expressing GFP-2×PH^{Osh2}, prepared under the same culture, buffer, and lysis conditions as in Fig. 3K (Methods). A post-nuclear supernatant generated by centrifugation at 1,000 × g for 5 min at 4°C was used as the Total fraction (T). Aliquots of T were centrifuged at 20,000 × g or 100,000 × g for 20 min at 4°C to obtain supernatant (S) and pellet (P) fractions; pellets were resuspended in HSE buffer. C. Distribution of GFP-2×PH^{Osh2} between supernatant (S) and pellet (P) fractions, expressed as the fraction of total (T) (data are shown as mean ± SD from three independent experiments; each dot represents one independent experiment).

Fig. S3

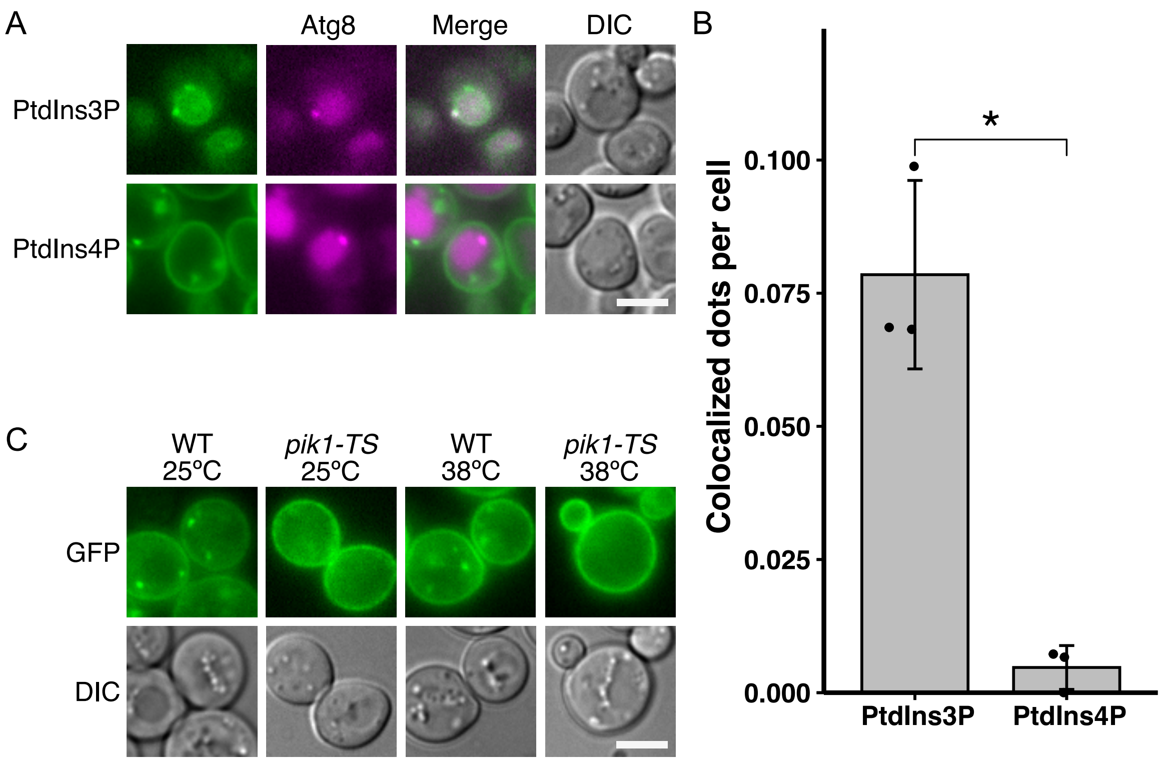

Fig. S3. Localization of GFP-2×PH^{Osh2} in WT and *pik1-TS* cells

A. *mRuby3-ATG8* cells carrying a GFP-2×PH^{Osh2} or GFP-2×FYVE (a PtdIns3P-specific probe) expression plasmid were grown to mid-log phase and treated with rapamycin for 1 hr. Scale bar: 4 µm. B. Quantification of GFP-2×PH^{Osh2} or GFP-2×FYVE and mRuby3-Atg8 colocalized dots per cell (data are shown as mean ± SD from three independent experiments; each dot represents one independent experiment; n > 50 cells per group; unpaired, two-tailed Student's t-test; *$P < 0.05$). Exact P values for all statistical comparisons are listed in Table S1. C. Cells carrying a GFP-2×PH^{Osh2} expression plasmid were pre-incubated at 25°C or 38°C for 30 min, followed by rapamycin treatment for 1 hr. Scale bar: 4 µm.
